# Repeated-Sprint Training in Hypoxia Induced by Voluntary Hypoventilation at Low Lung Volume: A Meta-analysis

**DOI:** 10.1186/s40798-025-00853-6

**Published:** 2025-05-16

**Authors:** Camille Précart, Janne Bouten, Xavier Woorons, Charly Fornasier-Santos, Grégoire P. Millet, Franck Brocherie

**Affiliations:** 1 University of Paris Cité, Paris, France; 2 Laboratory Sport, Expertise and Performance (EA 7370), French Institute of Sport (INSEP), Paris, France; 3 Univ. Lille, Univ. Artois, Univ. Littoral Côte d’Opale, ULR 7369 - URePSSS - Unité de Recherche Pluridisciplinaire Sport Santé Société, Lille, France; 4 Association for Research and Promotion of Hypoventilation Training (ARPEH), Lille, France; 5 Institute of Sport Sciences (ISSUL), Faculty of Biology and Medicine, Lausanne, Switzerland

## Abstract

**Background:**

Repeated-sprint training in hypoxia (RSH) induced through voluntary hypoventilation at low lung volume (RSH-VHL) may represent a low-cost alternative to systemic hypoxia. This meta-analysis aimed to systematically assess the effects of RSH-VHL training interventions on sea-level physical performance compared to similar repeated-sprint training with normal breathing (RSN).

**Methods:**

The PubMed/MEDLINE, SportDiscus^®^, ProQuest, and Web of Science online databases were examined from inception to February 2025 for original studies investigating the changes in physical performance following RSH-VHL and RSN. Only trained participants were included. Standardized mean difference (SMD) was determined for repeated-sprint ability related variables [*i.e.*, best and mean performance (RSA_best_ and RSA_mean_), sprint decrement score (S_dec_)] and maximal blood lactate concentration ([La]_max_). PEDro scale and Begg & Mazumbar test were used to assessed risk of bias.

**Results:**

From the 776 studies identified through systematic search, 10 studies including a total of 199 individuals (157 males and 42 females) were eligible for meta-analysis. While no effect was observed for RSA_best_ (SMD = 0.038; 95%CI − 0.252–0.328; P = 0.798) and RSA_mean_ (SMD = 0.276; 95%CI − 0.018–0.570; P = 0.066), moderate significant effects were observed for S_dec_ (SMD = 0.603; 95%CI 0.180–1.025; P = 0.005) and [La]_max_ (SMD = 0.611; 95%CI 0.223–0.999; P = 0.002) favoring RSH-VHL *vs*. RSN.

**Conclusion:**

Repeated-sprint training in hypoxia induced by voluntary hypoventilation at low lung volume provides putative gains in fatigue resistance during repeated sprints. Higher maximal blood lactate concentration suggests greater glycolytic contribution during RSH-VHL compared to RSN. Mechanisms underlying these effects are currently unclear and have yet to be identified.

## Key points

- The repeated-sprint training in hypoxia induced by voluntary hypoventilation at low lung volume (RSH-VHL) method has been proposed as an alternative to traditional repeated-sprint training in hypoxia (RSH), showing promising effects on physical performance enhancement, particularly repeated-sprint ability.
- This meta-analysis highlights that RSH-VHL can improve repeated-sprint fatigue resistance with a higher glycolytic contribution favoring RSH-VHL compared to similar training with normal breathing.
- For a better understanding of RSH-VHL underlying processes and to optimize training protocols, deeper investigation into potential mechanisms is warranted.

## Background

Over the last decade, repeated-sprint training in hypoxia (RSH) has gained popularity to improve sea-level physical performance, particularly repeated-sprint ability (RSA) [1–3]. This altitude/hypoxic training method targets microcirculatory skeletal muscle metabolic and molecular adaptations and promotes RSA enhancement [1, 4]. However, natural altitude or hypoxic devices are not always accessible to all athletes, limiting their opportunities to implement such specific hypoxic training method. Therefore, RSH induced by voluntary hypoventilation at low lung volume (RSH-VHL) was proposed as a “low-cost” alternative and recently integrated in the altitude/hypoxic training methods’ panorama [5]. RSH-VHL leads to hypoxemia, which may be due in part to a wider alveolar-to-arterial oxygen partial pressure difference, as a consequence of an increased ventilation-to-perfusion ratio inequality and a rightward shift of the oxygen dissociation under the effect of hypercapnia-induced acidosis [6, 7]. Hypoxemia induced by RSH-VHL is reflected through a decrease in peripheral capillary oxygen saturation (SpO_2_) ranging 88–91% [8, 9]. These values are comparable to the 84–89% measured during RSH performed at inspired oxygen fraction (FiO_2_) of 14.5%, equivalent to a simulated altitude of 3000 m [10, 11]. When performing RSH-VHL, participants have to first exhale down to the functional residual capacity and then hold their breath over an entire “all-out” sprint (generally ≤ 8 s) before breathing normally again during the subsequent recovery period (≤ 30 s), then repeat the process over 2 to 3 sets of 6–8 repetitions [8]. Beneficial RSH-VHL training-induced effects in either continuous supramaximal exercise [8] or RSA [12] have been reported. This method appears to be effective in both individual and team sports and could potentially be attributed to several factors. These include greater energy supply from anaerobic glycolysis supported by higher blood lactate production [8, 12] which may lead to gains in best (RSA_best_) and mean repeated-sprint performance (RSA_mean_). Additionally, higher oxygen uptake has been reported during repeated-sprint exercises and the following recovery periods after RSH-VHL [9] whereas greater muscle reoxygenation indicated by lower minimum deoxyhemoglobin concentration during recovery periods has also been found [13]. These two factors could enhance phosphocreatine resynthesis, promote greater metabolite elimination, thereby reducing RSA performance impairment and improving fatigue resistance*.*

Understanding the magnitude of the effects of RSH-VHL interventions is important to provide practical recommendations for practitioners. Therefore, the present study aimed to meta-analyze the available evidences on the effects of RSH-VHL *vs*. similar training with normal breathing (RSN) on sea-level physical performance.

## Methods

### Literature Search

This study was conducted in accordance with the ‘Preferred Reporting Items for Systematic Reviews and Meta-analyses’(PRISMA) guidelines [14]. A systematic search of the literature was conducted in four online databases (*i.e.*, PubMed/MEDLINE, SportDiscus®, ProQuest, and Web of Science) to find original interventional studies investigating the effects of RSH-VHL on sea-level physical performance with normal breathing. The search was restricted to “English language” and research articles published in peer-reviewed journals up to February 2025. The following terms were searched for in “all field”: [“hypoventilation OR reduced breath frequency OR breath holding” AND “exercise OR “training” NOT “obes* OR syndrome OR deficiency”]. This study was not pre-registered.

### Inclusion and Exclusion Criteria

To compare and quantify the difference between RSH-VHL and RSN, the following inclusion criteria were considered: (1) randomized controlled trial (*i.e.*, with at least a RSN control group); (2) trained participants (*i.e.*, regular training load > 4 h.week^−1^); (3) training intensity classified as “all-out”, “maximal”, “supramaximal” or “ > 150% maximal aerobic velocity (MAV) or power (MAP)”; (4) sprint duration ≤ 15 s and inter-sprint recovery ≤ 30 s; (5) intervention duration ≥ 2 weeks and (6) physical performance. Exclusion criteria were: (1) unhealthy, sedentary, or animal participants; (2) other hypoxic training methods.

### Data extraction

**Fig. 1:**
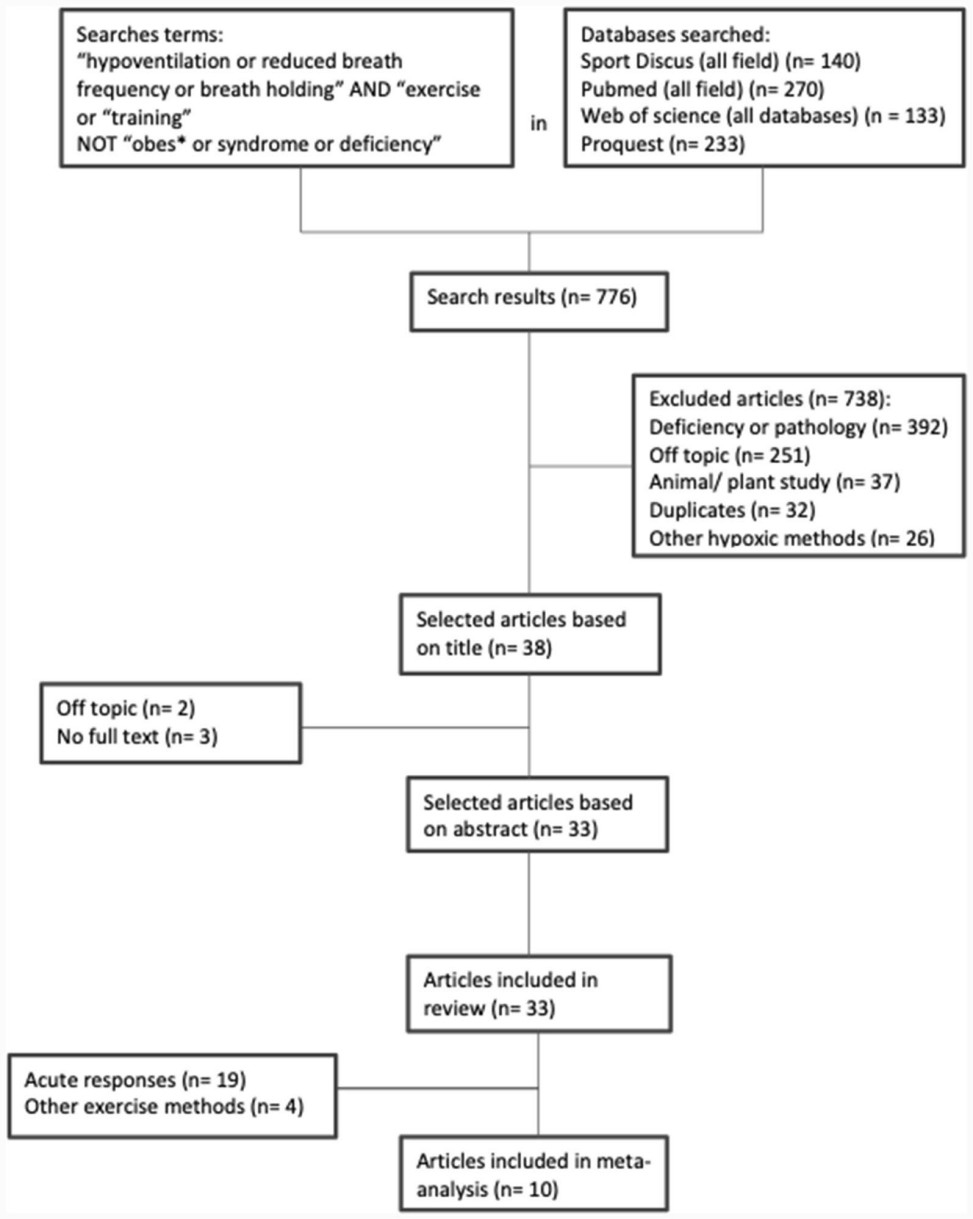
Flow chart of study selection

The systematic search on the databases revealed 776 studies (Fig. 1). Based on the removal of duplicates and screening of the title or abstract carried out by 2 investigators (CP and FB), 743 articles were further dismissed. A total of 33 full-text articles were evaluated, corresponding to all RSH-VHL studies including those limited to comparison of acute responses to a single RSH-VHL *vs*. RSN session, from which ten were eligible for meta-analysis. Each article was read and coded for the following descriptive variables: subjects, sex, training status, exercise mode, and training protocol. Sample size and physical performance data (mean and standard deviation [SD]) for both RSH-VHL and RSN pre- (baseline) and post-training intervention were extracted directly from text and tables or from figures using online graph digitizing software, PlotDigitizer (https://plotdigitizer.com/app, Porbital 2024, Phoenix, US*),* of the selected studies when only plots were published. Intermediate data (*i.e.*, between pre- and post- intervention) were not considered. Dependent variables included: RSA_best_ (fastest sprint time converted in velocity or highest power output), RSA_mean_ (averaged sprint time converted in velocity or power output), sprint decrement score (S_dec_) as an indicator of fatigue [15, 16], maximal blood lactate concentration ([La]_max_) for RSA tests and MAV or MAP for aerobic field tests [*e.g*., Yo-Yo Intermittent Recovery Test Level 1(YYIR1), 30–15 Intermittent Fitness Test (V_IFT_)].

### Data Analysis

#### Assessment of Reporting Quality and Risk of Bias

Assessment of reporting quality and risk of bias were evaluated by 2 investigators (CP and FB). Quality of publications was assessed with the Physiological Evidence Database (PEDro) scale. Due to the inherent characteristics of RSH-VHL, it is not feasible to implement blinding. Consequently, certain items of the PEDro scale—namely items 3, 5, 6 and 7, which involve concealing group allocations and blinding all subjects, therapists and assessors—were excluded, resulting in a reduced scale consisting of seven items only. The certainty of evidence for the meta-analyses of four variables was assessed using the GRADE framework [17]. This evaluation was based on five key factors—study limitations, inconsistency, imprecision, indirect evidence, and publication bias—following the related checklist [18] classifying the evidence as high, moderate, low, or very low.

#### Meta-analysis

The data collected were meta-analyzed with Comprehensive Meta-Analysis Software (version 4, Biostat, Inc., Englewood, NJ, USA) to provide the standardized mean difference (SMD) between the effects of RSH-VHL and RSN on sea-level physical performance via a random-effects model [19]. The use of the SMD as effect sizes were interpreted according to Cohen’s conventional criteria, with SMDs of < 0.2, 0.2–0.5, 0.5–0.8 and ≥ 0.8 representing trivial, small, moderate and large effect sizes, respectively [20]. Heterogeneity was determined using the *I*^2^ value (proportion of variance between studies that can be attributed to true variation in effect sizes rather than sampling error), with values of 25, 50 and 75 indicating low, moderate and high heterogeneity, respectively [19]. Study characteristics are presented as mean ± SD. Potential publication biases were accessed using Begg and Mazumdar’s rank correlation with asymmetry examination of funnel plots. We considered a P value < 0.05 as statistically significant. A “leave-one-out” sensitivity analysis was performed to assess the impact of each study on the overall effect estimate [21]. This approach tested the robustness of the meta-analysis findings by systematically recalculating the pooled effect size after excluding each study one at a time. A study was identified as an outlier if the “leave-one-out” pooled effect size fell outside the 95% confidence interval (95% CI) of the original pooled effect size.

## Results

### Study Characteristics and Publication Biases

Participant and training characteristics are displayed in Table 1. Five studies included only male participants [9, 22–25] while four others included both male and female participants [8, 12, 13, 26] and one study included only female participants [27]. Out of a total of 157 male and 42 female participants, studies included 20 ± 6 participants (10 ± 2 for RSH-VHL and 10 ± 3 for RSN), with an average age of 22 ± 7 years, height of 176 ± 6 cm, and weight of 73 ± 12 kg.

**Table 1:** Participants and studies characteristics

Studies	Participants [M, F; (RSH-VHL, RSN)]	Level	Sport	Intervention (weeks × sessions/week)	Mode	Training (sets × reps; intra-set and inter-set recovery)	Performance tests
Lörinczi et al. [25]	M (10, 10)	Semi-professional	Soccer	6 × 2	Running	2–3 × 6–8 × 50 m (“all-out”); 32–52 s intra-set recovery, 5–6 min active inter-set recovery	n × 30 m – ~ 25-s recovery threshold at 90% reference velocity (open-loop) (at least 10 sprints required)
Woorons et al. [26]	M (10), F (10); (10, 10)	International-national	Judo	4 × 2	Rowing	2–3 × 10–12 × ≈10 s (“all-out”); 20 s intra-set recovery, 3 min active inter-set recovery	8 × 25 s–25 s recovery
Ait Ali Braham et al. [27]	F (8, 8)	Trained	Soccer	6 × 2	Running	2–3 × 8–9 × 30 m (“all-out”); 25 s intra-set recovery, 5 min passive inter-set recovery	n × 30 m–25 s recovery voluntary cessation (open-loop)
Brocherie et al. [22]	M (16, 19)	International-national	Ice hockey	5 × 2	Running	2 × 6–8 × 40 m (“all-out”); 30-s intra-set recovery, 3 min semi-active inter-set recovery	12 × 40 m–30 s recovery
Fornasier-Santos et al. [24]	M (11, 10)	National	Rugby	4 × 2 (7 sessions)	Running	2–3 × 6–8 × 40 m (“all-out”); 22–25 s intra-set recovery, 3-min semi-active inter-set recovery	n × 40 m–24-s recovery threshold at 85% reference velocity (open-loop)
Lapointe et al. [13]	M (12), F (5); (9, 8)	National	Basketball	4 × 2	Running	3 × 6–8 × 6 s (“all-out”); 24-s intra-set recovery, 3 min semi-active inter-set recovery	12 × 30 m–20 s recovery V_IFT_
Trincat et al. [12]	M (9), F (7); (8, 8)	Regional-national	Swimming	2 × 3	Swimming	2 × 16 × 15 m (“all-out”); 30 s intra-set recovery, 20 min active inter-set recovery	n × 25 m–20 s recovery threshold at 94% reference velocity (open-loop)
Woorons et al. [8]	M(12), F (4); (8, 8)	Departmental-Regional	Triathlon	5 × 2	Swimming	12–20 × 25 m (200 m speed); 10–15-s intra-set recovery	400 m at maximal speed 200 m “all-out” 100 m “all-out”
Woorons et al. [9]	M (9, 9)	Regional-national	Cycling	3 × 2	Cycling	2–3 × 6–8 × 6 s (“all-out”); 24-s intra-set recovery, 3 min active inter-set recovery	10 × 6 s–30 s recovery Wingate
Woorons et al. [23]	M (10, 10)	Well-trained	Team sports	3 × 2	Cycling	3 × 8–12 × 8 s; (150% MAP) 24-s intra-set recovery, 3 min semi-active inter-set recovery	12 × 20 m–20 s recovery 200-m “all-out” running test YYIR1

*F* females, *M* males, *MAP* maximal aerobic power, *RSH-VHL* repeated-sprint training in hypoxia induced by hypoventilation at low lung volume, *RSN* repeated-sprint training in normoxia, *V*_*IFT*_ maximal velocity reached in the 30–15 Intermittent Fitness Test, *YYIR1* Yo-Yo test level 1

Training comprised running [13, 22, 24, 25, 27], cycling [9, 23], swimming [8, 12] and rowing [26]. Physical performance tests used the same exercise mode, with the exception of Woorons et al. [23] who specifically investigated the transferability of cycling training on overground RSA performance.

The RSH-VHL intervention lasted 2 to 6 weeks with 6 to 12 training sessions. Training protocols consisted of 1 to 3 sets of 6 to 20 sprint repetitions of 5 to 20 s duration interspersed with 10 to 52 s inter-sprint recovery and 3 to 20 min of inter-set rest. RSA tests differed in terms of number of repetitions [from 8 to 12 sprints in closed-loop tests (*i.e.*, with a constant number of sprints) or up to 85, 90 or 94% of reference velocity or voluntary cessation in open-loop tests (*i.e.*, task failure declared when peak velocity or power output dropped below 94%, 90% or 85% of the reference velocity or power output [12, 24, 25], or by voluntary cessation [27]), resulting to 9 to 23 sprints performed], sprint distance or duration (*i.e.*, from 20 to 50 m for running, 6 s for cycling and 25 m for swimming), as well as recovery duration and mode (*i.e.*, from 20 to 30 s, active or passive, respectively) (Table 1).

PEDro scores are presented in Table 2. Five studies showed weaker scores (6/7) because some participants were unable to complete the protocol (*i.e.*, injury or illness, unavailability, club transfer, or missed training sessions) [8, 13, 22, 24, 25, 27]. The five other studies correctly validated the seven items [9, 12, 23, 26]. Visual examination of the funnel plots and Begg and Mazumdar rank correlation test (P ≥ 0.19) did not indicate the presence of potential publication bias for the SMDs in RSA_best_, RSA_mean_, S_dec_, and [La]_max_. Biases' evaluation was impossible for MAV because of the insufficient number of studies (n = 2). Since the included studies were randomized controlled trials, the initial level of evidence was considered high a priori [17]. However, study limitations (lack of blinding) and imprecision (small sample sizes) led to a downgrade to moderate certainty for all variables. Despite low to moderate heterogeneity, the certainty of evidence remains moderate for S_dec_, likely due to a balance with an upgraded factor (moderate effect size).

**Table 2:**
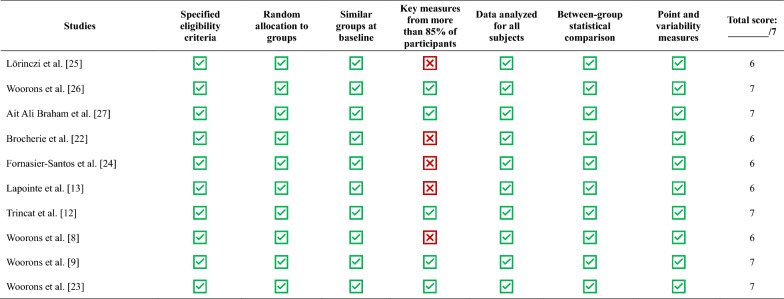
Quality assessment by Physiological Evidence Database scale (PEDro)

Key performance and physiological outcomes for each study and each group (RSH-VHL and RSN) are presented in Table 3.

**Table 3:** Studies training outcomes and testing results

Studies	Group (n)	HR_mean_ (bpm) SpO_2mean_ (%)	Performance changes (%)	Physiological changes (%)
Lörinczi et al. [25]	RSH-VHL (10 M)	– 78.5^##^	RSA_best_ (m.s^−1^) + 0.4 RSA_mean_ (m.s^−1^) + 2.9* S_dec_ − 2.2 Number of sprints + 75.3* YYIR2 + 18.3*	–
	RSN (10 M)	– 95.3^##^	RSA_best_ (m.s^−1^) + 0.2 RSA_mean_ (m.s^−1^) + 1.1* S_dec_ − 1.0 Number of sprints + 17.1 YYIR2 + 17.3*	–
Woorons et al. [26]	RSH-VHL (5 M, 5F)	– 78.7^##^	RSA_best_ (W) + 1.4 RSA_mean_ (W) + 3.6 S_dec_ − 3.5*	VO_2_/HR at rest + 17.6*
	RSN (5 M, 5F)	– 96.6^##^	RSA_best_ (W) + 3.0 RSA_mean_ (W) + 1.4 S_dec_ − 0.7	VO_2_/HR at rest + 10.2
Ait Ali Braham et al. [27]	RSH-VHL (8F)	– 92.1^#^	RSA_best_ (m.s^−1^) + 0.6 RSA_mean_ (% reference velocity) + 2.4* S_dec_ − 4.3* Number of sprints + 17.1*	–
	RSN (8F)	– 97.8^#^	RSA_best_ (m.s^−1^) + 0.9 RSA_mean_ (% reference velocity) + 0.2 S_dec_ − 0.3 Number of sprints + 0.5	–
Brocherie et al. [22]	RSH-VHL (16 M)	174^#^ 88.8	RSA_best_ (% reference velocity) + 1.0 RSA_mean_ (% reference velocity) + 4.0* S_dec_ − 4.0*	HR + 2.2*
	RSN (19 M)	175^#^ 95.8	RSA_best_ (% reference velocity) − 1.2 RSA_mean_ (% reference velocity) + 1.7 S_dec_ − 1.7	HR + 2.7*
Fornasier-Santos et al. [24]	RSH-VHL (11 M)	172 90.1	RSA_best_ (% reference velocity) + 0.5 RSA_mean_ (% reference velocity) − 0.7 Number of sprints + 63.7*	[La]_max_ − 8.0
	RSN (10 M)	176 95.5	RSA_best_ (% reference velocity) − 0.9 RSA_mean_ (% reference velocity) − 1.3 Number of sprints + 6.1	[La]_max_ − 21.5*
Lapointe et al. [13]	RSH-VHL (6 M, 3F)	– 87.7^#^	RSA_best_ (s) + 1.3 RSA_mean_ (s) − 2.5 S_dec_ -1.8* V_IFT_ + 7.5	[La]_max_ − 7.5
	RSN (6 M, 2F)	– 96.9^#^	RSA_best_ (s) + 1.0 RSA_mean_ (s) − 3.3 S_dec_ − 0.6 V_IFT_ + 6.7	[La]_max_ − 10.2
Trincat et al. [12]	RSH-VHL (4 M, 4F)	160^#^ 94.6^#^	RSA_best_ (m.s^−1^) + 1.1 RSA_mean_ (m.s^−1^) + 1.8 S_dec_ + 0.2 Number of sprints + 35.2* Reference velocity (m.s^−1^) + 1.7*	[La]_max_ + 39.8*
	RSN (5 M, 3F)	166^#^ 97.7^#^	RSA_best_ (m.s^−1^) + 1.7 RSA_mean_ (m.s^−1^) + 3.0 S_dec_ − 0.5 Number of sprints + 8.8 Reference velocity (m.s^−1^) + 1.7*	[La]_max_ + 10.9
Woorons et al. [8]	RSH-VHL (6 M, 2F)	154 90,7	400 m time − 4.4* 200 m time − 3.6* 100 m time − 3.5*	[La]_max_ + 42.2* + 24.6* + 41.5*
	RSN (6 M, 2F)	157 98,5	400 m time + 0.14 200 m time − 0.4 100 m time− 0.04	[La]_max_ − 2.4 + 4.6 + 1.7
Woorons et al. [9]	RSH-VHL (9 M)	– 87.7	RSA_best_ (W) + 5.9 RSA_mean_ (W) + 7.7* S_dec_ − 4.1* Wingate (W) + 4.8*	Wingate [La]_max_ + 15.7* VO_2_ + 26.3* at exercise; + 20.9 at rest*
	RSN (9 M)	– 95.6	RSA_best_ (W) + 2.7 RSA_mean_ (W) + 2.2 S_dec_ − 1.4 Wingate + 0.5	Wingate [La]_max_ − 6.3 VO2 + 2.7 at exercise; − 0.5 at rest
Woorons et al. [23]	RSH-VHL (10 M)	– 87.6	RSA_best_ (m.s^−1^) no change RSA_mean_ (m.s^−1^) + 1.8 S_dec_ − 2.5* YYIR1 + 32.1* 200-m time − 1.5	[La]_max_ + 5.1
	RSN (10 M)	– 96.2	RSA_best_ (m.s^−1^) + 0.5 RSA_mean_ (m.s^−1^) + 0.5 S_dec_ − 0.2 YYIR1 + 4.0 200-m time − 0.4	[La]_max_ no change

*F*: females, *HR* heart rate, *M* males, *[La]*_*max*_ maximal blood lactate concentration (mmol.L^−1^), *RSA*_*bes*t_ best repeated-sprint ability performance, *RSA*_*mean*_ mean repeated-sprint ability sperformance, *RSH-VHL* repeated-sprint training in hypoxia induced by hypoventilation at low lung volume, *RSN* repeated-sprint training in normoxia, *S*_*dec*_ sprint decrement score, *SpO*_*2mean*_ mean peripheral capillary oxygen, *VO*_*2*_ oxygen consumption, *YYIR1* Yo-Yo intermittent recovery test, level 1, *V*_*IFT*_ maximal velocity reached in the 30–15 intermittent fitness *test*

^*^Significant change from pre- to post-test

^#^Mean value included recovery

^##^Mean minimal value per sprint

### Meta-analysis

**Fig. 2:**
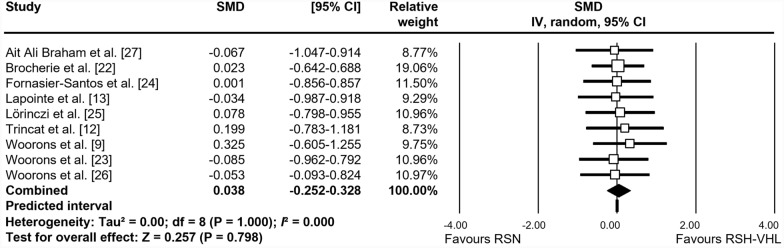
Forest plot of the standardized mean difference (SMD) between the effect of repeated-sprint training in hypoxia induced by voluntary hypoventilation at low lung volume (RSH-VHL *vs*. similar training with normal breathing (RSN) on best RSA performance (RSA_best_)

**Fig. 3:**
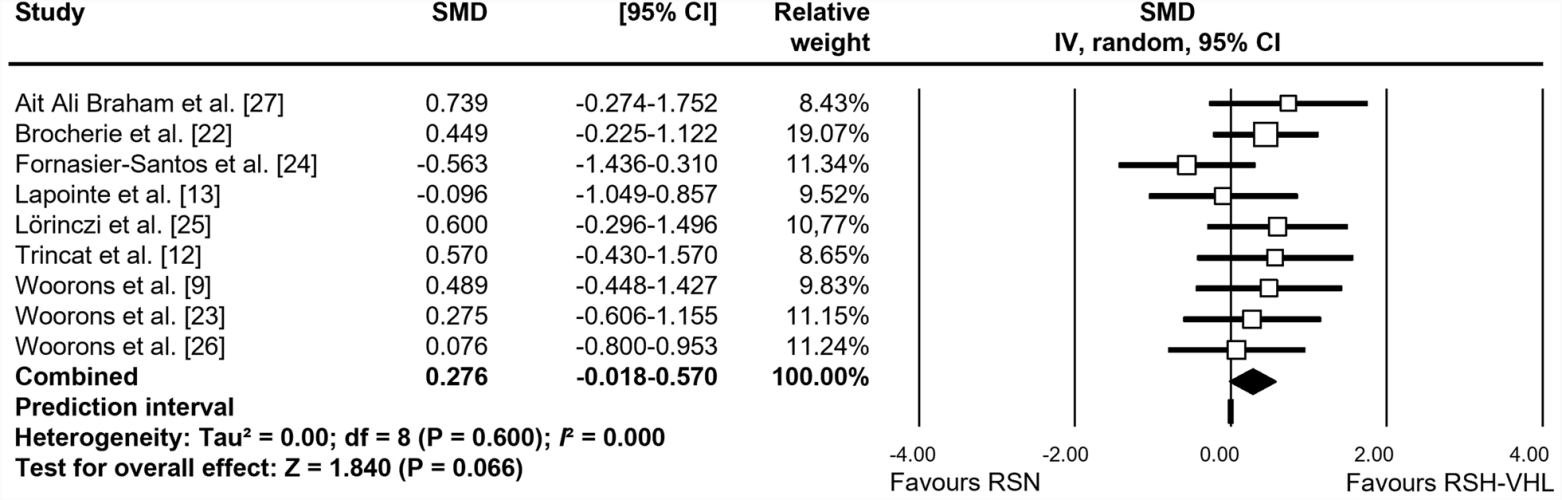
Forest plot of the standardized mean difference (SMD) between the effect of repeated-sprint training in hypoxia induced by voluntary hypoventilation at low lung volume (RSH-VHL *vs*. similar training with normal breathing (RSN) on mean RSA performance (RSA_mean_)

**Fig. 4:**
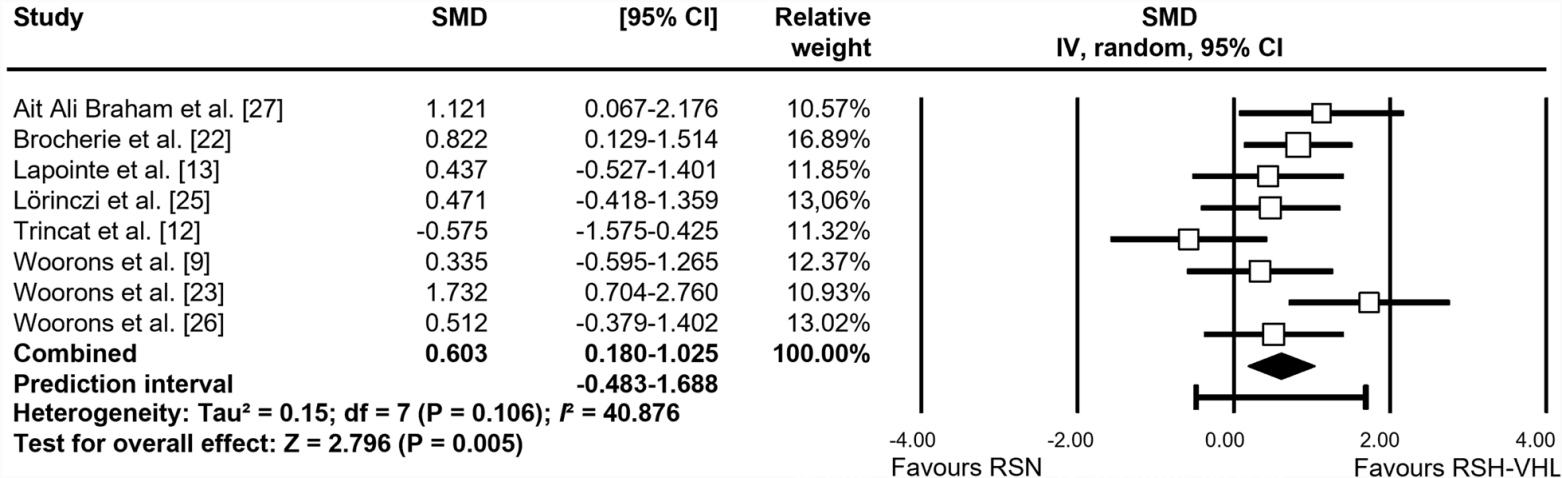
Forest plot of the standardized mean difference (SMD) between the effect of repeated-sprint training in hypoxia induced by voluntary hypoventilation at low lung volume (RSH-VHL *vs*. similar training with normal breathing (RSN) on sprint decrement score (S_dec_)

**Fig. 5:**
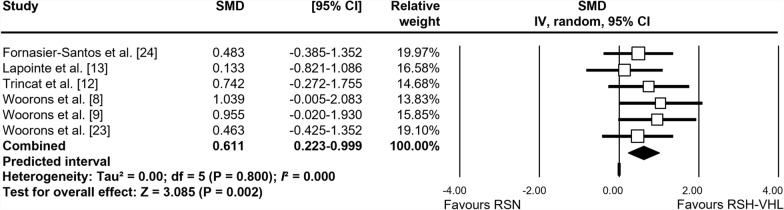
Forest plot of the standardized mean difference (SMD) between the effect of repeated sprint training in hypoxia induced by voluntary hypoventilation at low lung volume (RSH-VHL *vs*. similar training with normal breathing (RSN) on maximal blood lactate ([La]_max_)

The forest plots depicting the individual SMD and associated 95% CI and random-effects model for RSA_best_, RSA_mean_, S_dec_, and [La]_max_ are shown in Figs. 2, 3, 4 and 5, respectively. After training, RSA_best_ did not differ between RSH-VHL and RSN (SMD = 0.038, 95% CI -0.252 to 0.328; *trivial* effect; P = 0.798; Fig. 2). While no significant effect was found for RSA_mean_ (SMD = 0.276; 95% CI -0.018 to 0.570; *small* effect; P = 0.066; Fig. 3), S_dec_ (SMD = 0.603; 95% CI − 0.180 to 1.025; P = 0.005; Fig. 4) and [La]_max_ (SMD = 0.611; 95% CI -0.223 to 0.999; P = 0.002; Fig. 5) showed significant *moderate* effects for RSH-VHL compared with RSN. Random-effects model could not be performed for MAV due to insufficient number of studies (n = 2).

Heterogeneity was not detected among studies assessing RSA_best_ (*I*^*2*^ = 0.0%), RSA_mean_ (*I*^*2*^ = 0.0%) and [La]_max_ (*I*^*2*^ = 0.0%), while it was low to moderate for S_dec_ (*I*^*2*^ = 40.9%). The “leave-one-out” sensitivity analysis revealed no outlier for any variables.

## Discussion

This meta-analysis aimed to evaluate the effects of RSH-VHL *vs*. RSN training interventions on sea-level physical performance, particularly on RSA. The aggregated findings revealed significantly greater gains in S_dec_, with higher glycolytic contribution in RSH-VHL compared to RSN. On the contrary, improvement in RSA_best_ and RSA_mean_ did not differ between RSH-VHL and RSN.

A positive significant effect was revealed for S_dec_ favoring RSH-VHL compared to RSN, meaning higher fatigue resistance. A previous study recommended S_dec_ as the most suitable indicator of fatigue because it considers data from each sprint and includes both RSA_best_ and RSA_mean_ variables [16]. Although not significant when meta-analyzing the data, these two variables benefit from RSH-VHL and participate in the significant effect observed for S_dec_ in favor of RSH-VHL. For RSH-VHL, this moderate enhancement in RSA fatigue resistance could be partly related to the moderate increase in [La]_max_ compared to RSN, suggesting an enhancement of the glycolytic contribution that may result from both VHL-induced hypercapnic acidosis [7] and hypoxic stress and/or increased acute anaerobic glycolytic activity [8]. This may be particularly beneficial during repeated-sprint exercises, in which the anaerobic glycolysis is one of the most limiting factors. Indeed, it supplies approximately 40% of the total energy during a single 6-s sprint, with a progressive inhibition of glycolysis as sprints are repeated [15]. While the enhancement in glycolytic activity and pH regulation have been demonstrated in systemic RSH [1], it is currently unknown to which extent RSH-VHL induced hypoxia (with lower hypoxic dose compared to systemic RSH) and/or additional hypercapnia promote greater glycolytic adaptations. Indeed, hypercapnia leads to respiratory acidosis, which accentuates hypoxemia and muscle deoxygenation during VHL [28] and RSH-VHL methods [7, 29]. This may contribute to higher lactate release during recovery [28]. Conversely, systemic hypoxia, while also inducing an increase in anaerobic contribution, triggers a hypoxic ventilatory response leading to the opposite effect, hypocapnia. Meanwhile, the direct role of hypercapnia or hypocapnia in blood lactate production appears to be controversial. The more substantial hypoxic stress in systemic RSH could also be responsible to provoke a greater glycolytic response, as reflected by the higher [La]_max_ during acute RSH compared to RSH-VHL [30].

By meta-analyzing RSA_best_, the present study showed no significant effect for this variable after RSH-VHL compared to RSN. When taken individually, 7 studies including RSA_best_ demonstrated a positive increase although only 4 studies reported a trivial non-significant enhancement after intervention compared to RSN (+ 1.8% favoring RSH-VHL). Repeated-sprint training per se seems to provide conflicting results on RSA_best_ [31], possibly due to variations in protocols (sprint duration, recovery, exercise-to-recovery ratio). For instance, in normoxic RSA protocols, longer sprint durations and shorter exercise-to-recovery ratios (*e.g.*, 1:5) may be more effective to further stimulate the anaerobic glycolysis and enhance single-sprint performance [31]. Conversely, recent RSH studies suggest that sprint durations up to 10 s [32] and high exercise-to-recovery ratios (*e.g.*, 1:3) [33, 34] should be used for maximizing hypoxic stress benefits. In this view, RSH-VHL-induced hypoxic stress may be reduced with excessively long recovery periods or short exercise-to-recovery ratios, as the first sprint generally does not induce desaturation [29]. While traditional RSH (*e.g.*, [2, 35, 36]) seems to be more effective than RSN, the present findings did not demonstrate a significant enhancement of RSA_best_ with the RSH-VHL method. This is likely due to the difficulty for producing “all-out” velocity or power when focusing on correct breathing VHL techniques and maintaining breath-holds, compared to systemic hypoxia. Additionally, despite similar SpO_2_ levels induced either by RSH-VHL or RSH during sprints, systemic hypoxia induces stronger hypoxic dose as this method provides a longer duration and more severe hypoxic stress [30], leading to more pronounced muscle deoxygenation that persists during recovery.

Surprisingly, no significant effect was observed either for RSA_mean_. The contradicting effects of RSH-VHL on RSA_mean_ may arise from the use of closed- (*i.e*., with a fixed number of sprints) or open-loop (*i.e*., with a variable number of sprints determined by a fatigue threshold) protocols which alter the calculation of RSA_mean_. This is likely explained by protective pacing measures occurring during open-loop “all-out” sprints, despite strong verbal encouragement when the task endpoint is unknown [37]. For example, using an open-loop RSA test, Fornasier-Santos et al. [24] and Trincat et al. [12] reported an increase in the number of sprints performed after RSH-VHL intervention (respectively 15 and 10 sprints compared to 9 and 7 sprints before the intervention), with a large inter-individual variability in decreasing sprint performance. This variability could be influenced by individual fatigue resistance [38], linked to differences in neuromuscular and energetic profiles of participants, and highly associated with the reference velocity and phosphagen system [39]. Such RSH-VHL-induced improvement in either RSA_mean_ in closed-loop RSA tests or in the number of sprints performed in open-loop protocols may be related to greater muscle (re)oxygenation as previously demonstrated with systemic RSH [1]. Furthermore, a notable enhancement in sprint-by-sprint performance is predominantly evident during the latter phase of the RSA test, specifically when considering RSA_mean_ dataset within close-loop protocols (pre- to post-intervention changes of + 1.4% in the first part of RSA test [from 1 to 5-10th sprint] to + 5.4% in the second part [from 7–11 to 10-19th sprint] for RSH-VHL *vs*. + 0.1% to + 2.0% for RSN). Average oxygen consumption during an RSA test turned out to be higher after RSH-VHL but not after RSN [9]. The VHL-derived “pump” effect, resulting from the deep and brief inspirations combined with breath-holds, might potentially increase ventricular diastolic filling and thus stroke volume. In an acute study, Woorons et al. [6] revealed that oxygen uptake during intra-set recovery was approximately 6% higher than in normoxia, likely due to the “pump” effect.

Considering the aforementioned potential increase in oxygen uptake during exercise, RSH-VHL may therefore induce enhancements in aerobic tests. The latest RSH-VHL study reported a lower heart rate and higher oxygen uptake/heart rate during the recovery periods of the RSA test, which could suggest an increase in stroke volume due to the potential “pump effect” [26]. However, this assumption is insufficient to explain improvements in maximal oxygen consumption and maximal aerobic continuous test. It may, however, contribute to improvements in intermittent aerobic tests, as it could enhance recovery by increasing oxygen availability [40]. Two studies reported a significant improvement in intermittent aerobic tests (YYIR1 and V_IFT_) [13, 23]. However, due to the limited number of studies, no meta-analysis could be performed for this variable. More studies are needed to confirm whether RSH-VHL intervention is effective to generate improvement in MAV (or MAP) compared to RSN.

The present results should be interpreted with caution due to the low number of studies, which reduces the strength of the meta-analysis. Variation in protocols, training, and testing modes could introduce bias in the comparability of studies. The low to moderate heterogeneity observed could be explained by the disparate results in the included studies, particularly the differences between open- and closed-loop repeated-sprint protocols. Additionally, even with accurate indicators [16], the relationship between fatigue levels and performance is not always straightforward, as it highly depends on RSA_best_ and RSA_mean_ [15]. Moreover, none of the studies were conducted with blinding of either participants or assessors due to the voluntary nature of the VHL method, so we cannot rule out a reduced sensitivity to risk of bias. But even removing some PEDro items, all studies are deemed as “high quality studies” (*i.e.*, above cut-off values of 6) [41]. Additionally, despite the inclusion of studies exclusively involving females, the proportion of female participants remains low compared to the total number of participants in the meta-analysis (21.1%). Further studies with more female participants are warranted to ensure that the same evidences apply to female athletes and to investigate potential sex-based physiological differences in RSH-VHL-induced adaptation.

## Practical Applications

The benefits from RSH and RSH-VHL have been demonstrated for a large range of sports [2, 3]. In comparison to RSH, RSH-VHL may induce a different oxidative-glycolytic balance known for being influenced to a large extent by the sprint duration and the exercise-to-recovery ratio [32, 33]. While the optimal combination of RSH-VHL and RSH sessions in order to target the glycolytic pathway and the buffering capacity remains unknown, one may speculate that it could be an effective method in intermittent sports, particularly those requiring a high lactate production.

Another point to consider is the exercise mode that may be crucial to induce central and peripheral adaptations. The physiological adaptations that enhance anaerobic glycolysis after RSH-VHL occur within the muscle tissue and are therefore highly exercise-dependent for improving RSA [9, 12]. In this context, it appears to be more effective to train using the same exercise mode as in competition, targeting the same muscle groups. Moreover, Woorons et al. [7] suggested that swimming might be less effective in inducing hypoxic stress, due to the horizontal position, leading to a more homogeneous ventilation-perfusion ratio, which diminishes the alveolar-arterial oxygen gradient. Nevertheless, more recent studies show similar levels of SpO_2_ with swimming mode training [9, 12].

However, the adaptations following RSH-VHL are not limited to peripheral pathways, as Woorons et al. [23] suggested a potential improvement in stroke volume associated with the “pump” effect. As this mechanism occurs at the central level, transferability also appears possible, especially for enhancing aerobic tests and RSA fatigue-induced resistance [23]. Nevertheless, mastering a correct breathing technique is necessary to lead to such adaptations. An exercise mode with a seated position and only lower limb activation such as cycling could be recommended for novice practitioners to learn how to exhale correctly before breath hold and maintain it until the end of the sprint. A nose clip could be worn if the participant is unable to close the glottis to prevent the air coming from the nasal ways to reach the lungs.

## Perspectives

The RSH-VHL protocols reported here mostly followed the recommendations suggested for the traditional systemic RSH method [2], except for Trincat et al. [12] who used 20 min of inter-set recovery. However, maintaining breath-hold during maximal effort presents a limitation in the variation of sprint duration. Extending sprint duration beyond 8 s or equivalent distance becomes very demanding. Furthermore, Woorons et al. [9] used active recovery for inter-set recovery but passive one for intra-set recovery. In most repeated-sprint training interventions (either RSH or RSN), passive recovery is generally preferred as it can delay exhaustion [42]. Whether adopting different recovery modes improve or alter RSH-VHL sprinting performance remains to be explored.

RSH-VHL appears as a valid, cost-effective alternative to traditional systemic RSH, with improvements of RSA_best_ (1–6% for RSH-VHL *vs*. 5–10% for RSH), RSA_mean_ (4–8% for RSH-VHL *vs*. 10–11% for RSH), and the number of sprints performed (35–75% for RSH-VHL *vs*. 38–57% for RSH). However, to the best of our knowledge, no study has yet compared the chronic effects of RSH-VHL *vs*. systemic RSH on sea-level physical performance. By comparing the acute physiological responses of RSH-VHL *vs*. systemic RSH, Imai et al. [30] showed that RSH (3000 m) induced a greater muscle deoxygenation than RSH-VHL but did not induce hypercapnia when performing 3 × 6 × 8 s at 170% of maximal oxygen consumption, with 16 s of active recovery at 30% of maximal oxygen consumption between sprints and 3 min of passive rest between sets. Prolonging end-expiratory breath holding up to the breaking point leads to more severe deoxygenation [43], and seems to provide additional benefits for improving RSA with RSH-VHL [25, 26]. Whether such method would induce similar adaptations than systemic RSH remains to be investigated. Further research is warranted to understand the RSH-VHL- *vs*. systemic RSH-underlying mechanisms leading to sea-level physical performance improvement. This will be crucial to determine which training protocol to incorporate in sport season scheduling.

## Conclusion

The present meta-analysis indicates that a training intervention based on repeated-sprint training in hypoxia induced by voluntary hypoventilation at low lung volume provides putative gains in fatigue resistance and glycolytic energy supply compared to similar training with normal breathing. These effects were unclear for best and mean repeated-sprint performance, although this could be related to the methodology used in three studies (open- *vs*. closed-loop tests) but were significant for the fatigue decrement score. In order to optimize training protocols, in-depth investigation into potential underlying mechanisms is required.

## Data Availability

Data are available from the corresponding author on reasonable request.

## Abbreviations

FiO_2_: Inspired oxygen fraction
[La]_max_: Maximal blood lactate concentration
MAP: Maximal aerobic power
MAV: Maximal aerobic velocity
RSA: Repeated-sprint ability
RSA_best_: Best RSA performance
RSA_mean_: Mean RSA performance
RSH: Repeated-sprint training in hypoxia
RSH-VHL: RSH induced by voluntary hypoventilation at low lung volume
RSN: Repeated-sprint training with normal breathing
S_dec_: Sprint decrement score
SpO_2_: Peripheral capillary oxygen saturation
SMD: Standardized mean difference
V_IFT_: 30–15 Intermittent Fitness Test
YYIR1: Yo-Yo Intermittent Recovery Test Level 1
